# Rigorous Analysis and Systematical Design of Double-Layer Metal Superlens for Improved Subwavelength Imaging Mediated by Surface Plasmon Polaritons

**DOI:** 10.3390/nano12203553

**Published:** 2022-10-11

**Authors:** Jing Wang, Zhichao Li, Weina Liu

**Affiliations:** 1 Costar (Shanghai) Science & Technology Co., Ltd., Shanghai 200241, China; 2 Institute of Advanced Optics, China South Industries Group Corporation, Nanyang 473000, China; 3 Costar Group Co., Ltd., Nanyang 473000, China; 4 Nanyang Lida Optic-Electronics Co., Ltd., Nanyang 473000, China

**Keywords:** superlens, subwavelength imaging, optical transfer function, planar waveguide, surface plasmon polaritons

## Abstract

A double-layer metal superlens was rigorously analyzed and systematically designed to improve subwavelength imaging ability. It was revealed that transmission properties of the imaging system could be accurately interpreted by the five-layer waveguide mode theory—each amplification peak among the spatial frequency range of evanescent waves was associated with a corresponding surface plasmon polariton (SPP) mode of an insulator-metal-insulator-metal-insulator (IMIMI) structure. On the basis of such physical insight, evanescent waves of higher spatial frequency were effectively amplified via increasing propagation constants of symmetrically coupled short-range SPP (s-SRSPP) and antisymmetrically coupled short-range SPP (a-SRSPP), and evanescent waves of lower spatial frequency were appropriately diminished by approaching to cut off symmetrically coupled long-range SPP (s-LRSPP). A flat and broad optical transfer function of the imaging system was then achieved, and improved subwavelength imaging performance was validated by imaging an ideal thin object of two slits with a 20-nm width distanced by a 20-nm spacer, under 193-nm illumination. The resolution limit of the designed imaging system with double-layer superlens was further demonstrated to be at least ~λ/16 for an isolated two-slit object model. This work provided sound theoretical analysis and a systematic design approach of double-layer metal superlens for near-field subwavelength imaging, such as fluorescent micro/nanoscopy or plasmonic nanolithography.

## 1. Introduction

The diffraction limit, as a fundamental obstacle to producing high-resolution images, emerges because high-spatial-frequency evanescent waves carrying information on the fine details of objects decay exponentially. In 2000, Pendry theoretically proposed that, for TM polarized light, under the electrostatic limit, a planar metal layer with negative permittivity and a positive permeability can be used for subwavelength imaging beyond the diffraction limit [1]. The underlying principle is to compensate for exponential decay of evanescent fields away from the object by amplifying evanescent waves. The metal superlens with subwavelength imaging ability was later demonstrated experimentally with 60 nm half-pitch resolution at the UV frequency range by Zhang et al. in 2005 [2], on the condition that the metal slab and the surrounding dielectrics have a matched permittivity. Since then, great progress [3,4,5,6,7,8,9,10] has been made for super-resolution imaging by metal planar superlens.

For single metal planar superlens, different approaches have been proposed to improve near-field subwavelength imaging performance. An intuitive fashion is to reduce losses of metal superlens. As a low-loss metal in the DUV range, a single aluminum (Al) superlens was investigated both theoretically and experimentally [11]. Besides, the use of gain media [12] and diluted metals [13] has been suggested to lower losses. It was also demonstrated that subwavelength imaging can be achieved via reflection instead of transmission from the metal slab, which was less susceptible to metal material loss [14]. Another way of improving subwavelength imaging of single superlens is to design and optimize the structural parameters of the imaging system. In Zhang’s experiment, 35-nm thick Ag superlens was adopted with an optimum transfer function, since thinner superlens showed higher but narrower enhancement bands, and thicker ones provided smaller enhancements [15]. Moore et al. suggested an Ag superlens system with a total thickness of over 120 nm in order to obtain a flat transfer function in photolithography [16]. Sheng et al. proposed balancing the amplification of evanescent waves and the flatness of the transfer function with a designed unmatched permittivity condition [17,18] for thin superlens. However, all these schemes with a single superlens mainly amplified evanescent waves of relatively lower spatial frequency.

To amplify evanescent waves over a broad range of spatial frequencies, multilayer superlenses with alternative metal-dielectric layers have been proposed [5,8,19,20,21,22,23,24,25]. It was indicated that the multilayer system was relatively robust to losses and could enhance higher-spatial-frequency evanescent waves to improve imaging resolution beyond the diffraction limit. Nevertheless, the optical transfer function of a multilayer system usually showed one or more sharp enhancement band(s) due to multiple Fabry-Pérot (F-P) resonance [19,20,21,22]. Besides, the anisotropic permittivity of a multilayer system was usually calculated by effective medium theory (EMT) [5,8,21,22,23,24], which became less accurate when the variant layer thickness was not small enough compared with wavelength [26,27]. Actually, it was also reported that a multilayer system may not provide a better imaging performance than a few-layer superlens system [28].

Double-layer metal superlens for subwavelength imaging has also attracted attention [29,30,31] because their imaging performance is superior to single superlens system, whilst their structure and fabrication is much simpler than a multilayer superlens system. Blaikie et al. experimentally compared [29] double-layer planar superlens with single-layer superlens in lithography and concluded that the resolution limit of a double-layer superlens was as good as or better than a single layer for the same total metal thickness. Elsayad et al. theoretically investigated [30] the imaging ability of a bilayer planar superlens for imaging a fluorescent dye based on the approximate EMT and indicated there was a regime where the plasmonic amplification at the metal-dielectric surfaces together with the canalization-like effects from the anisotropy of the structure produced favorable conditions for sub-resolution imaging. Luo et al. studied [31] a surface plasmon interference nanolithography technology with a double-layer planar silver lens. They treated two Ag superlenses with a finite thickness (10 nm to 50 nm) and a sandwiched Al_2_O_3_ layer as a three-layer semi-infinite metal-insulator-metal (MIM) waveguide supporting up to two surface plasmon polariton (SPP) modes, which is unsound, especially for an Ag thickness approaching 10 nm. Due to a lack of accurate and sound theoretical analysis, the mentioned designs of the double-layer metal superlens imaging system had less physical ground [29,30,31].

In this article, an imaging system with a double-layer metal superlens was rigorously analyzed and systematically designed to improve subwavelength imaging performance. We regarded it as three cascaded F-P cavities—two superlens cavities and a sandwiched dielectric cavity—and calculated the optical transfer function of the imaging system by considering multiple reflections inside these cavities. Meanwhile, the imaging systems was seen as a five-layer insulator-metal-insulator-metal-insulator (IMIMI) waveguide structure, and up to four surface plasmon polariton (SPP) modes could be obtained by numerically solving the implicit dispersion relation of the IMIMI waveguide. We revealed that the transmission properties of imaging system with double-layer metal superlens could be accurately explained by the five-layer waveguide mode theory—each amplification peak (up to four peaks) among the spatial frequency range of evanescent waves was associated with a corresponding SPP mode (up to four modes) of the IMIMI structure. Based on such physical insight, with a full exploration of the double-layer metal superlens imaging structure, the evanescent waves of higher spatial frequency were effectively amplified via increasing propagation constants of symmetrically coupled short-range SPP (s-SRSPP) and antisymmetrically coupled short-range SPP (a-SRSPP), and evanescent waves of lower spatial frequency were appropriately diminished by approaching to cut off symmetrically coupled long-range SPP (s-LRSPP), to form a flat and broad transfer function of the imaging system. The improved subwavelength imaging performance by double-layer metal superlens was validated by imaging an ideal thin object of two slits with a 20-nm width, distanced by a 20-nm spacer, under 193-nm illumination, and the resolution limit of the designed imaging system was further verified to reach at least ~λ/16 for an isolated two-slit object.

## 2. Near-Field Imaging System of Double-Layer Metal Superlens

**Figure 1:**
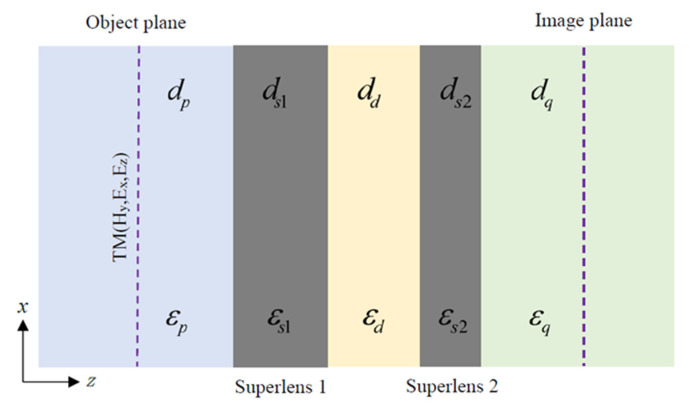
Near-field subwavelength imaging system of double-layer metal superlens.

The imaging system consists of a double-layer metal superlens with permittivities $\varepsilon_{s1}$ and $\varepsilon_{s2}$, separated by a dielectric layer with permittivity $\varepsilon_{d}$. Their corresponding thickness is $d_{s1}$, $d_{s2}$ and $d_{d}$, respectively. This finite-thickness metal-insulator-metal (f-MIM) structure [32,33,34] is sandwiched by two semi-infinite dielectric media, with permittivities $\varepsilon_{p}$ and $\varepsilon_{q}$, as shown in Figure 1, which is actually a five-layer insulator-metal-insulator-metal-insulator (IMIMI) waveguide structure [35,36,37,38]. A virtual object plane in the dielectric medium $\varepsilon_{p}$ at a distance $d_{p}$ from the superlens $\varepsilon_{s1}$ represents object fields without considering the specific forms of incident sources. The image plane is placed in a semi-infinite dielectric medium $\varepsilon_{q}$ at a distance $d_{q}$ from the superlens $\varepsilon_{s2}$. The object fields in the medium $\varepsilon_{p}$ could excite surface plasmon polaritons along the interface of the IMIMI structure. Both the propagating waves and the SPP waves will reach the image plane and contribute to the image in a dielectric medium $\varepsilon_{q}$.

### 2.1. Optical Transfer Function

The imaging system shown in Figure 1 can be regarded as three cascaded F-P cavities. The first cavity is the superlens layer $\varepsilon_{s1}$ with two metal/dielectric interfaces, $\varepsilon_{s1}|\varepsilon_{p}$ and $\varepsilon_{s1}|\varepsilon_{d}$, respectively. The second cavity is the dielectric layer with two interfaces, $\varepsilon_{d}|\varepsilon_{s1}$ and $\varepsilon_{d}|\varepsilon_{s2}$, respectively. The third cavity is a metal superlens layer $\varepsilon_{s2}$ with two metal/dielectric interfaces, $\varepsilon_{s2}|\varepsilon_{d}$ and $\varepsilon_{s2}|\varepsilon_{q}$, respectively. The transmission coefficient of the metal superlens cavity of permittivity $\varepsilon_{s1}$ can be calculated [1] as follows:

(1) $$\tau_{s1}=\frac{e_{s1}\cdot t_{p\_s1}\cdot t_{s1\_d}}{1-e_{s1}^{2}\cdot r_{s1\_p}\cdot r_{s1\_d}}$$

where the Fresnel reflection and transmission coefficients from medium *m* to medium *n*, with sub-indices *m (, n )* = *p, s1, d, s2* and *q*, are $r_{m\_n}=\left(\varepsilon_{n}k_{z\_m}-\varepsilon_{m}k_{z\_n}\right)/\left(\varepsilon_{n}k_{z\_m}+\varepsilon_{m}k_{z\_n}\right)$, $t_{m\_n}=2\varepsilon_{n}k_{z\_m}/\left(\varepsilon_{n}k_{z\_m}+\varepsilon_{m}k_{z\_n}\right)$ with $k_{z\_m(,n)}=\sqrt{\varepsilon_{m(,n)}k_{0}^{2}-k_{x}^{2}}$; the exponential factor $e_{m(,n)}=\exp\left(ik_{z\_m(,n)}d_{m(,n)}\right)$, describing the phase change of the propagating waves with $k_{x}^{2}<\varepsilon_{m(,n)}k_{0}^{2}$ along the distance $d_{m(,n)}$ and the exponent decay in the amplitude of the evanescent waves $k_{x}^{2}>\varepsilon_{m(,n)}k_{0}^{2}$ over $d_{m(,n)}$, respectively. In the same manner, the transmission coefficient of the metal superlens cavity of permittivity $\varepsilon_{s2}$ is expressed by the following:

(2) $$\tau_{s2}=\frac{e_{s2}\cdot t_{d\_s2}\cdot t_{s2\_q}}{1-e_{s2}^{2}\cdot r_{s2\_d}\cdot r_{s2\_q}}$$

For a dielectric F-P cavity of permittivity $\varepsilon_{d}$ sandwiched by a double-layer metal superlens, its transmission coefficient can be calculated [39,40,41] by considering the recursive reflections in the cavity as follows:

(3) $$\tau_{d}=\frac{e_{d}}{1-e_{d}^{2}\left(\frac{r_{d\_s2}+e_{s2}^{2}\cdot r_{s2\_q}}{1-e_{s2}^{2}\cdot r_{s2\_d}\cdot r_{s2\_q}}\right)\left(\frac{r_{d\_s1}+e_{s1}^{2}\cdot r_{s1\_p}}{1-e_{s1}^{2}\cdot r_{s1\_p}\cdot r_{s1\_d}}\right)}$$

As the f-MIM structure is a cascade of three F-P cavities, the total transmission coefficient $\tau_{f-MIM}$ is calculated by the following:

(4) $$\tau_{f-MIM}=\tau_{s1}\cdot \tau_{d}\cdot \tau_{s2}$$

The optical transfer function of the imaging system from the object plane to the image plane is defined as the transmission coefficient for the spatial wavevector components of object fields along the interfaces of superlenses, and can be written as follows:

(5) $$OTF\left(k_{x}\right)=e_{p}\cdot \tau_{f-MIM}\cdot e_{q}.$$

According to Equations (4) and (5), for propagating waves, there is only a phase difference between $\tau_{f-MIM}$ and OTF, and thus $\left|OTF\right|=\left|\tau_{f-MIM}\right|$, while for for evanescent waves, $\left|OTF\right|$ is smaller than $\left|\tau_{f-MIM}\right|$ due to the exponential delay factors in Equation (5).

### 2.2. Waveguide Dispersion Equation

The imaging system of double-layer metal superlens can be treated as the finite-thickness MIM waveguide structure [32,33,34], or a five-layer semi-infinite IMIMI waveguide structure [35,36,37,38], instead of the three-layer semi-infinite MIM waveguide structure Luo et al. claimed [31]. The waveguide modes of IMIMI (f-MIM) are quite different from that of a three-layer semi-infinite MIM waveguide. According to Maxwell’s equations, together with continuity boundary conditions of tangential electric fields and magnetic fields in the IMIMI waveguide structure, a set of equations of SPP mode fields (E_x_, H_y_, E_z_) can be expressed [42,43,44], which leads to a transcendental dispersion equation in an implicit form as follows [45]:

(6) $$e_{d}^{2}=\frac{e_{s2}\left(\frac{k_{z\_q}}{\varepsilon_{q}}-\frac{k_{z\_s2}}{\varepsilon_{s2}}\right)\left(\frac{k_{z\_q}}{\varepsilon_{q}}-\frac{k_{z\_s2}}{\varepsilon_{s2}}\right)+e_{s2}^{-1}\left(\frac{k_{z\_q}}{\varepsilon_{q}}+\frac{k_{z\_s2}}{\varepsilon_{s2}}\right)\left(\frac{k_{z\_q}}{\varepsilon_{q}}+\frac{k_{z\_s2}}{\varepsilon_{s2}}\right)}{e_{s2}\left(\frac{k_{z\_q}}{\varepsilon_{q}}-\frac{k_{z\_s2}}{\varepsilon_{s2}}\right)\left(\frac{k_{z\_q}}{\varepsilon_{q}}+\frac{k_{z\_s2}}{\varepsilon_{s2}}\right)+e_{s2}^{-1}\left(\frac{k_{z\_q}}{\varepsilon_{q}}+\frac{k_{z\_s2}}{\varepsilon_{s2}}\right)\left(\frac{k_{z\_q}}{\varepsilon_{q}}-\frac{k_{z\_s2}}{\varepsilon_{s2}}\right)}\;\cdot \frac{e_{s1}\left(\frac{k_{z\_p}}{\varepsilon_{p}}-\frac{k_{z\_s1}}{\varepsilon_{s1}}\right)\left(\frac{k_{z\_s1}}{\varepsilon_{s1}}-\frac{k_{z\_d}}{\varepsilon_{d}}\right)+e_{s1}^{-1}\left(\frac{k_{z\_p}}{\varepsilon_{p}}+\frac{k_{z\_s1}}{\varepsilon_{s1}}\right)\left(\frac{k_{z\_s1}}{\varepsilon_{s1}}+\frac{k_{z\_d}}{\varepsilon_{d}}\right)}{e_{s1}\left(\frac{k_{z\_p}}{\varepsilon_{p}}-\frac{k_{z\_s1}}{\varepsilon_{s1}}\right)\left(\frac{k_{z\_s1}}{\varepsilon_{s1}}+\frac{k_{z\_d}}{\varepsilon_{d}}\right)+e_{s1}^{-1}\left(\frac{k_{z\_p}}{\varepsilon_{p}}+\frac{k_{z\_s1}}{\varepsilon_{s1}}\right)\left(\frac{k_{z\_s1}}{\varepsilon_{s1}}-\frac{k_{z\_d}}{\varepsilon_{d}}\right)}$$

By computationally searching the solution, the complex propagation constant $\beta =k_{x}$, the effective mode index $n_{eff}=\beta /k_{0}=k_{x}/k_{0}$ and field profiles of the SPP modes supported by the IMIMI waveguide could be obtained.

It is helpful to regard the IMIMI waveguide as two coupled IMI waveguides [43]. In the limit of $d_{d}\to \infty$, two IMI waveguides are totally uncoupled. Each independent IMI usually supports a long-range SPP (LRSPP) at lower spatial frequencies and a short-range SPP (SRSPP) at higher spatial frequencies [17,41]. When two IMI waveguides get close enough, mode fields begin to couple symmetrically or anti-symmetrically, which would result in up to four SPP modes—symmetrically coupled LRSPP (s-LRSPP), antisymmetrically coupled LRSPP(a-LRSPP), symmetrically coupled SRSPP (s-SRSPP), and antisymmetrically coupled SRSPP (a-SRSPP), depending on the specific parameter configuration of the IMIMI waveguide [33,35,43,45,46]. When $d_{d}=0$, the IMIMI becomes a single IMI waveguide with a metal thickness equal to the addition of two superlenses thickness.

### 2.3. Exemplary Imaging System with Initial Parameters

**Figure 2:**
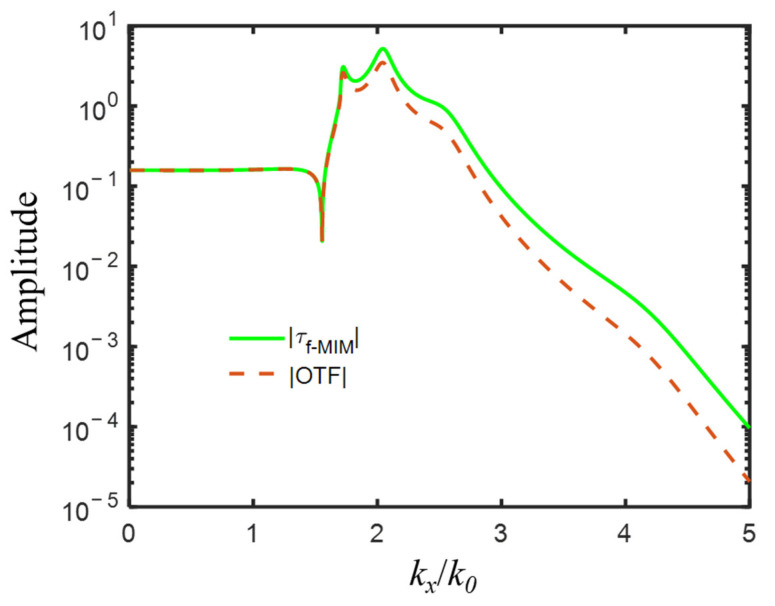
The amplitude of optical transfer function $\left|OTF\right|$ and transmission coefficient $\left|\tau_{f-MIM}\right|$ with four amplification peaks of evanescent waves, for imaging system with the following initial parameters: $\varepsilon_{s1}=\varepsilon_{s2}$ = −4.43 + *i*0.42, $\varepsilon_{p}$ = 2.4, $\varepsilon_{d}$ = 2.4, $\varepsilon_{q}$ = 2.89, $d_{s1}$ = 25 nm, $d_{s2}$ = 12 nm, $d_{p}$ = 5 nm, $d_{d}$ = 60 nm, $d_{q}$ = 5 nm.

For an exemplary imaging system of a double-layer superlens with the following initial parameters: 193-nm incident wavelength with TM(E_x_, H_y_, E_z_) polarization, two Al layers with permittivity $\varepsilon_{s1}=\varepsilon_{s2}$ = −4.43 + *i*0.42 [47], thickness $d_{s1}$ = 25 nm and $d_{s2}$ = 12 nm, the central dielectric layer $\varepsilon_{d}$ = 2.4 (SiO_2_) and $d_{d}$ = 60 nm, two semi-infinite dielectric $\varepsilon_{p}$ = 2.4 (SiO_2_) and $\varepsilon_{q}$ = 2.89 (photoresist), the amplitude of optical transfer function $\left|OTF\right|$ and transmission coefficient $\left|\tau_{f-MIM}\right|$ as a function of normalized wavevector $k_{x}/k_{0}$ was depicted in Figure 2, from which, we could notice that for propagation waves with $k_{x}/k_{0}<\sqrt{\varepsilon_{p}}=1.55$, $\left|OTF\right|=\left|\tau_{f-MIM}\right|<1$; for evanescent waves with $k_{x}/k_{0}>1.55$, $\left|OTF\right|<\left|\tau_{f-MIM}\right|$, and it was disproportionately amplified. Four amplification peaks were shown at $k_{x}/k_{0}$ = 1.72, 2.04, 2.55, and 4.20, among the spatial frequency range of evanescent waves. The first two peaks located at relatively low spatial frequency were relatively narrow and high, and the other two at relatively high spatial frequency were relatively broad and low.

**Figure 3:**
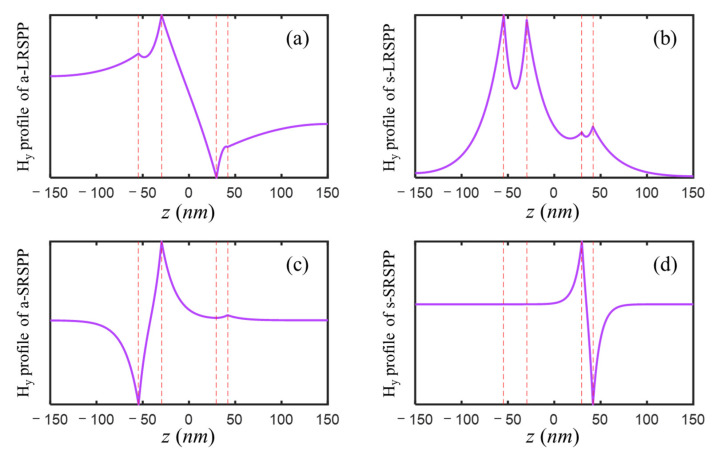
H_y_ profile of (**a**) a-LRSPP, (**b**) s-LRSPP, (**c**) a-SRSPP, and (**d**) s-SRSPP mode of five-layer IMIMI imaging structure with the initial parameters. The middle dielectric layer was centered at *z* = 0, and two superlenses were indicated by red dash lines.

For the IMIMI waveguide with the initial parameters listed above, the real parts of the solved effective mode indices $real\left(n_{eff}\right)=real\left(k_{x}/k_{0}\right)$ were 1.7174, 2.0432, 2.5532, and 4.2023, which well corresponded to the normalized frequency $k_{x}/k_{0}$ of four amplification peaks of $\left|OTF\right|$ and $\left|\tau_{f-MIM}\right|$, respectively. The a-LRSPP and s-LRSPP modes with effective indices of 1.717 and 2.043 accounted for the two narrow and high peaks, while a-SRSPP and s-SRSPP with mode indices of 2.553 and 4.202 contributed to the latter two broad and low peaks. The solved H_y_ profiles of the four modes supported by the IMIMI waveguide were plotted in Figure 3, among which, s-LRSPP in Figure 3b caused the maximum transmission at normalized wavevector 2.04 in Figure 2.

## 3. Improved Subwavelength Imaging by Double-Layer Metal Superlens

Since the introduction of a specific optical source would exert a profound influence on the imaging system [30,41,48,49,50], an ideal object of two slits [17,21,51] with a width of $w_{slit}=20\;\mathrm{nm}$ and center-to-center distance $d_{c-c}=40\;\mathrm{nm}$ along *x* direction was used as a simple and straightforward way for judging subwavelength imaging ability of double-layer metal superlens. The amplitude of object fields is 1 in the slits and 0 otherwise, and thus the spatial frequency of the ideal object could be expressed as follows:

(7) $$H_{object}\left(k_{x}\right)=2w_{slit}\frac{\sin\left(k_{x}w_{slit}/2\right)}{k_{x}w_{slit}/2}\cos\left(\frac{k_{x}d_{c-c}}{2}\right)$$

The fields in the imaging plane could then be obtained by inverse Fourier transformation as follows [26,51]:

(8) $$H_{image}\left(x\right)=\frac{1}{2\pi }\int \left[H_{object}\left(k_{x}\right)\cdot OTF\left(k_{x}\right)\cdot \exp\left(ik_{x}x\right)\right]dk_{x}$$

Now that the transmission properties of the imaging system of double-layer metal superlens were rigorously interpreted by the SPP mode theory of IMIMI waveguide, for optimizing the optical transfer function of the imaging system to improve the subwavelength imaging ability, the influence of structure parameters on effective indices of SPP modes was investigated.

### 3.1. Thickness of f-MIM

**Figure 4:**
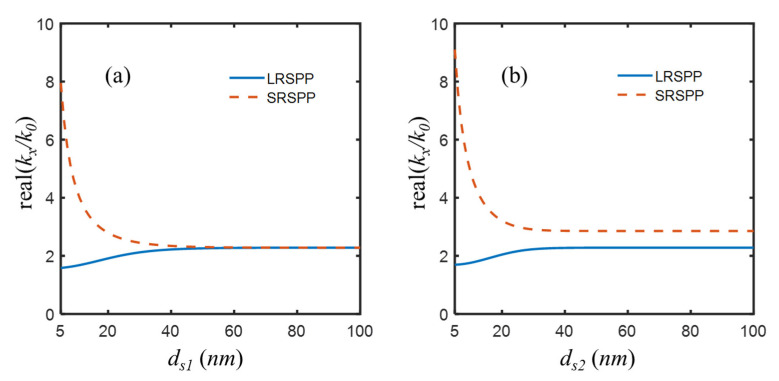
Real part of effective mode indices $n_{eff}=k_{x}/k_{0}$ of LRSPP and SRSPP supported by totally independent (**a**) symmetric IMI with $\varepsilon_{p}=\varepsilon_{d}$ = 2.4 and (**b**) asymmetric IMI with $\varepsilon_{p}$ = 2.4, $\varepsilon_{q}$ = 2.89, for Al superlens of thickness ranging from 5 nm to 100 nm, when the thickness of central dielectric $d_{d}$ of IMIMI is infinite.

The initial parameters were taken, except that the thickness of the central dielectric $d_{d}$ was changed to infinite to make the system become two totally independent IMI waveguides. For the IMI with a symmetric structure ($\varepsilon_{p}=\varepsilon_{d}$= 2.4), the effective indices of SPP modes are shown in Figure 4a, from which it was known that two modes, LRSPP and SRSPP, were supported when the Al superlens is thinner than 50 nm. As the thickness of the metal superlens ranged from 5 nm to 100 nm, the effective index of the LRSPP mode gradually grew from 1.5842 to 2.2818, while the effective index of SRSPP mode dramatically decreased from 7.9552 to 2.2821. When the Al superlens is thicker than 50 nm, the two modes were about to degenerate to one with an effective mode index of 2.282. For the IMI with an asymmetric structure ($\varepsilon_{d}$ = 2.4, $\varepsilon_{q}$ = 2.89), the effective index of the LRSPP mode increased from 1.6942 to 2.2819, and the effective index of the SRSPP mode decreased from 9.1018 to 2.8559. The two modes are not degenerate into one due to the asymmetry of the IMI structure, as shown in Figure 4b. Overall, thinner superlens could provide amplification for evanescent waves in a higher frequency range thanks to the SRSPP [52]. Therefore, providing fabrication difficulties, two Al layers of 5 nm thickness [53,54] were used, although technically, a thinner Al superlens, such as 2 nm [55], could be rather preferred.

**Figure 5:**
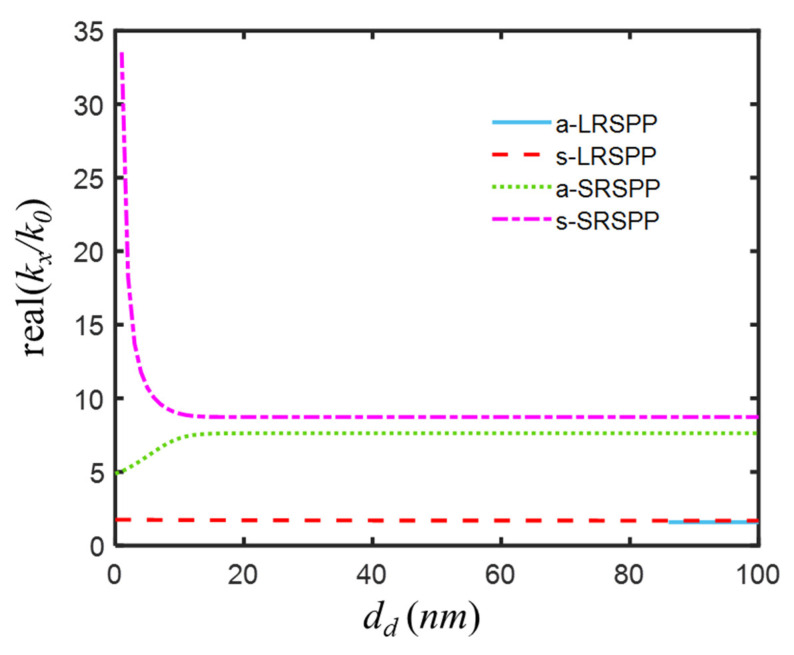
Real part of effective mode indices $n_{eff}=k_{x}/k_{0}$ of a-LRSPP, s-LRSPP, a-SRSPP, and s-SRSPP of IMIMI waveguide for variant thickness of central dielectric layer $d_{d}$ from 100 nm to 0 nm, with $d_{s1}=d_{s2}$ = 5 nm.

As two IMI waveguides got closer, mode fields began to couple. From Figure 5, it was known to us that four modes were supported when the thickness of the central layer was 100 nm, and the real parts of effective mode indices of a-LRSPP, s-LRSPP, a-SRSPP, and s-SRSPP of this weak coupling IMIMI waveguide were 1.5814, 1.6919, 7.6273, and 8.7287, respectively. When the thickness of the central layer continued to decrease, the a-LRSPP disappeared [42,46] at 85-nm thickness. The real part of the effective index of the a-LRSPP mode increased slightly from 1.6919 to 1.7611, while that of the a-SRSPP mode decreased from 7.6273 to 4.8850, as the central layer thickness changed from 100 nm to 0 nm. When the central layer thickness was 0 nm, the two modes were actually SPP modes supported by an asymmetric IMI with a 10-nm superlens in Figure 4b. The coupling effect became dramatic for the thin central layer (<10 nm), which made the propagation constant of s-LRSPP rapidly move to the high-frequency range. Therefore, a thinner (but nonzero) central dielectric layer was favorable to subwavelength imaging, such as a 5-nm central layer.

**Figure 6:**
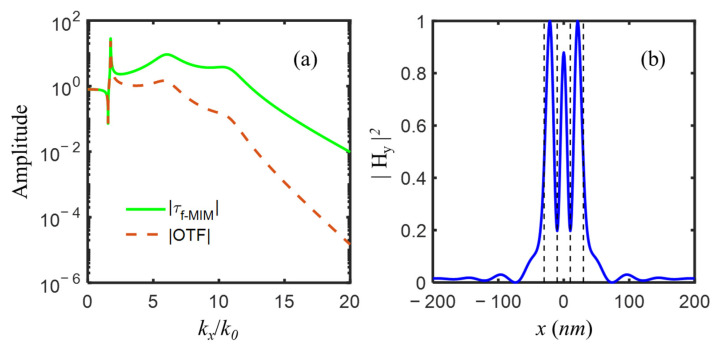
(**a**) The amplitude of optical transfer function $\left|OTF\right|$ and transmission coefficient $\left|\tau_{f-MIM}\right|$ with three amplification peaks of evanescent waves, and (**b**) the image of two-slit object represented by black dash lines, for imaging system with the following parameters: $\varepsilon_{s1}=\varepsilon_{s2}$ = −4.43 + *i*0.42, $\varepsilon_{p}$ = 2.4, $\varepsilon_{d}$ = 2.4, $\varepsilon_{q}$ = 2.89, $d_{s1}$ = $d_{s2}$ = $d_{p}$ = $d_{d}$ = $d_{q}$ = 5 nm.

The transmission coefficient $\tau_{f-MIM}$ of the f-MIM waveguide and optical transfer function OTF of the imaging system after f-MIM thickness optimization, e.g., Al(5 nm)/SiO_2_(5 nm)/Al(5 nm), was shown in Figure 6a. Three amplification peaks at $k_{x}/k_{0}$=1.7462, 6.0719, and 10.7102 of evanescent waves corresponded to s-LRSPP, a-SRSPP, and s-SRSPP, respectively. All these three modes contributed to the amplifications among a broad frequency range. The object of two 20 nm slits spaced 20 nm apart could be well resolved in the image plane as in Figure 6b, however, with an unwanted high central peak and two side lobes.

### 3.2. Refractive Index of Central Dielectric Layer

**Figure 7:**
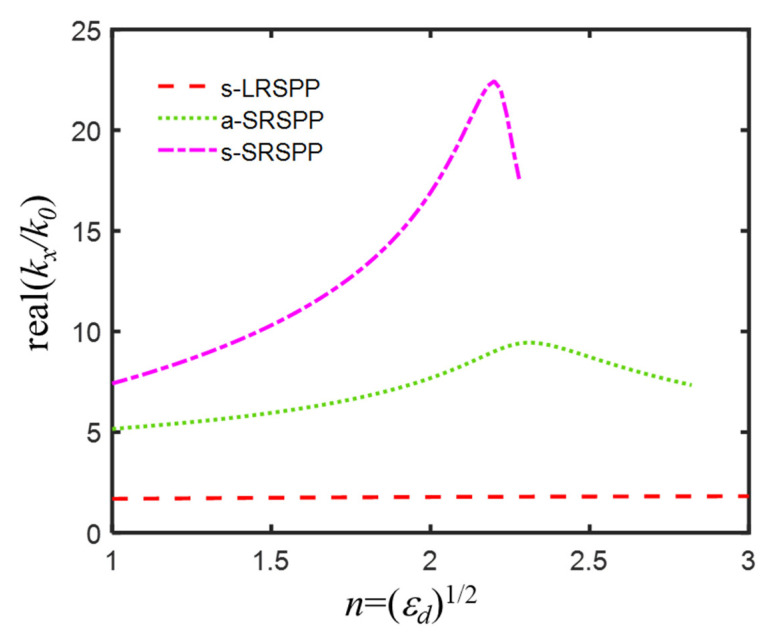
Real part of effective mode indices $n_{eff}=k_{x}/k_{0}$ of s-LRSPP, a-SRSPP, and s-SRSPP of IMIMI waveguide for variant refractive index $\sqrt{\varepsilon_{d}}$ of central dielectric layer from 1 to 3 with an increment of 0.02.

**Figure 8:**
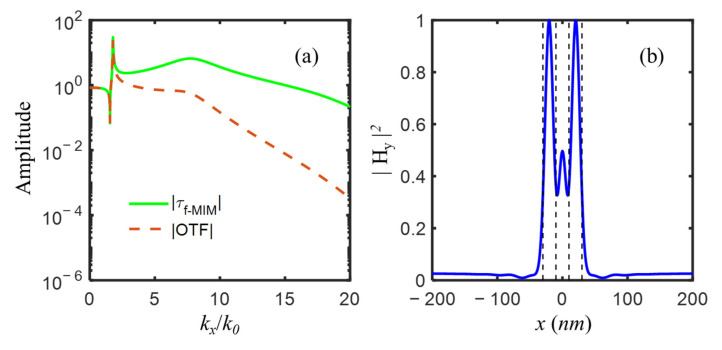
(**a**)The amplitude of optical transfer function $\left|OTF\right|$ and transmission coefficient $\left|\tau_{f-MIM}\right|$ with three amplification peaks of evanescent waves, and (**b**) the image of two-slit object represented by black dash lines, for imaging system with the following parameters: $\varepsilon_{s1}=\varepsilon_{s2}$ = −4.43 + *i*0.42, $\varepsilon_{p}$ = 2.4, $\varepsilon_{d}$ = 4.08, $\varepsilon_{q}$ = 2.89, $d_{s1}$ = $d_{s2}$ = $d_{p}$ = $d_{d}$ = $d_{q}$ = 5 nm.

For better image quality, the amplification of high spatial frequency was enhanced via changing the refractive index of the central dielectric layer, instead of using an alternately metal-dielectric multilayer structure. The effective indices of s-LRSPP, a-SRSPP, and s-SRSPP modes as a function of the variant refractive index of the central dielectric layer from 1 to 3 with an increment of 0.02 are plotted in Figure 7, from which it could be seen that s-LRSPP mode always existed with an effective index in proximity to 1.7, a-SRSPP mode index reached its maximum of 22.4353 as the central dielectric index $\sqrt{\varepsilon_{d}}$ increased to 2.2 from 1, and then rapidly reduced to 17.4327 when $\sqrt{\varepsilon_{d}}$ slightly increased to 2.28, above which a-SRSPP mode was cut-off. For s-SRSPP, it had the largest mode index of 9.4443 at $\sqrt{\varepsilon_{d}}$ = 2.32 and would disappear when the central dielectric index was larger than 2.82. Therefore, for amplification of evanescent waves among a broad spatial frequency range, it was suggested that the refractive index window of the central dielectric layer was chosen between 2 and 2.28. For robustness, magnesium oxide (MgO, with *n* = 2.02) [47], as a typical high-index material at 193 nm wavelength, was suggested to use for the central layer. The real parts of the mode indices of s-LRSPP, a-SRSPP, and s-SRSPP were 1.7805, 7.7989, and 17.3991 for IMIMI with MgO, corresponding to the three amplification peaks of evanescent waves in Figure 8a. By comparing Figure 8a with Figure 6a, it is evidenced that the amplification of evanescent waves with normalized spatial frequency $k_{x}/k_{0}$ ranging from 11 to 20 is further enhanced, almost with a magnitude of one order. The two slits were clearly distinguished in Figure 8b, in which the unwanted central peak and sidelobes were largely suppressed, compared with that in Figure 6b.

### 3.3. Refractive Index of Dielectric Layer before Double-Layer Superlens

**Figure 9:**
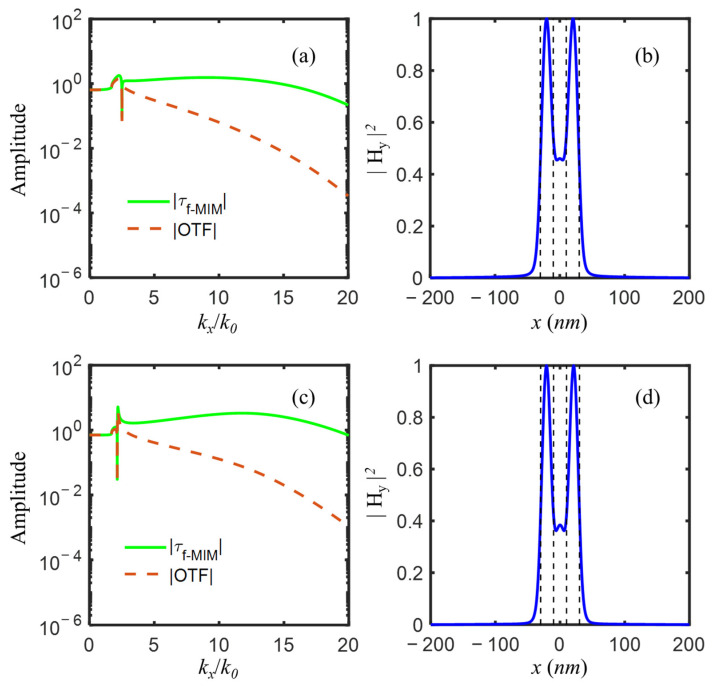
Completely cutoff s-LRSPP with $\varepsilon_{p}$ = 6.2 (**a**) The amplitude of optical transfer function $\left|OTF\right|$ and transmission coefficient $\left|\tau_{f-MIM}\right|$, and (**b**) the image of two-slit object represented by black dash lines; Approaching to cutoff s-LRSPP with $\varepsilon_{p}$ = 4.58 (**c**) The amplitude of optical transfer function $\left|OTF\right|$ and transmission coefficient $\left|\tau_{f-MIM}\right|$, and (**d**) the image of two-slit object represented by black dash lines, for imaging system with the following parameters: $\varepsilon_{s1}=\varepsilon_{s2}$ = −4.43 + *i*0.42, $\varepsilon_{d}$ = 4.08, $\varepsilon_{q}$ = 2.89, $d_{s1}$ = $d_{s2}$ = $d_{p}$ = $d_{d}$ = $d_{q}$ = 5 nm.

To further improve image ability, the amplitude of the optical transfer function was flattened by diminishing the remarkable sharp peak at a lower spatial frequency among the evanescent wave range in Figure 6a and Figure 8a. This amplification was associated with the s-LRSPP mode in Figure 5 and Figure 7, and was much higher than other transmission enhancements. To flatten the amplitude of the optical transfer function, the permittivity $\varepsilon_{p}$ of the dielectric layer (SiO_2_) before the double-layer superlens was changed to meet the cut-off condition [45] of s-LRSPP mode as follows: Imkz_q=0, which can be satisfied by Imkx=0 and 0<Rekx≤εqk0. The high peak at a lower spatial frequency of the optical transfer function was completely erased as shown in Figure 9a after cutting off the s-LRSPP mode with silicon carbide (SiC, with *n* = 2.49 at 193 nm) [56]; meanwhile, the image was further improved as shown in Figure 9b, since the unwanted center peak of the image was now almost completely removed, compared with the image shown in Figure 6b and Figure 8b.

Although the s-LRSPP cutoff method completely erased the sharp amplification peak at lower spatial frequency, it also decreased the amplification of evanescent waves at higher spatial frequency, which can be seen by comparing Figure 8a and Figure 9a. We then preferred to diminish the high peak by approaching the cutoff [17] s-LRSPP, rather than erase the high peak by completely cutoff s-LRSPP. As a commonly used material in semiconductor lithography, lutetium aluminum garnet (LuAG, with *n* = 2.14 at 193 nm) [57] was then used to replace SiC. As shown in Figure 9c, the amplification peak at a low spatial frequency of optical transfer function still existed but was largely diminished from the high peak in Figure 8a. Meanwhile, evanescent waves at high spatial frequency were more enhanced in Figure 9c than that in Figure 9a. From Figure 9d, it was known to us that approaching to cutoff s-LRSPP method produced a notably improved image with better contrast, compared with the image from the completely cutoff s-LRSPP method in Figure 9b.

**Figure 10:**
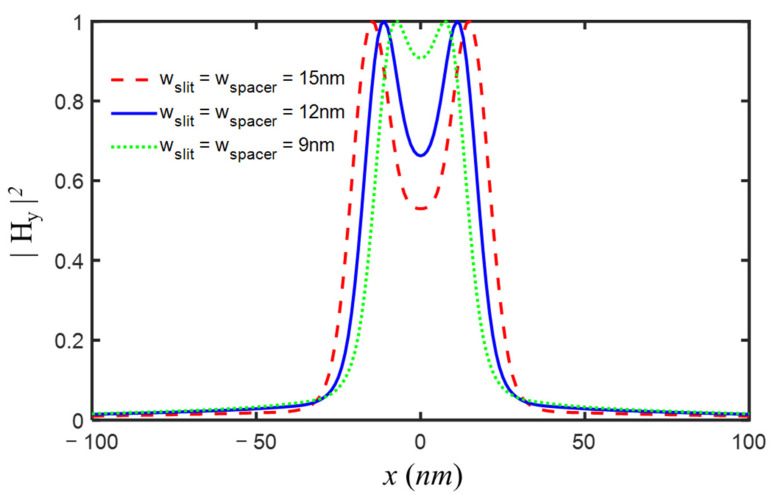
The images of two-slit object with slit width and spacer width of 15 nm, 12 nm, and 9 nm, for designed imaging system with the following parameters: $\varepsilon_{s1}=\varepsilon_{s2}$ = −4.43 + *i*0.42, $\varepsilon_{p}$ = 4.58, $\varepsilon_{d}$ = 4.08, $\varepsilon_{q}$ = 2.89, $d_{s1}$ = $d_{s2}$ = $d_{p}$ = $d_{d}$ = $d_{q}$ = 5 nm.

Although a two-slit model with a 20-nm slit width and a 20-nm slit spacer was used during the design process, the finer two-slit object could be resolved by the double-layer superlens imaging system we designed. The images of the two-slit object with (15 nm, 12 nm, or 9 nm) slit width and corresponding slit spacer were presented in Figure 10. Obviously, two 15-nm slits distanced by a 15-nm spacer were well differentiated, and the two slits with a 12-nm width and a 12-nm spacer could still be resolved, while the two-slit object with a 9-nm slit width and a 9-nm spacer was almost indistinguishable. Therefore, for the isolated two-slit model, the designed imaging system with double-layer superlens could reach a resolution limit of ~λ/16, at least.

## 4. Conclusions

We investigated the imaging performance of bilayer metal planar superlens for near-field subwavelength imaging, based on the transfer function and waveguide mode theory. The bilayer metal superlens imaging system was considered as a cascade of three F-P cavities, and the transfer function of the imaging system was then calculated by considering multiple reflections inside these three cavities, which showed that evanescent waves were amplified disproportionately with several peaks. At the same time, the imaging system was seen as a five-layer insulator-metal-insulator-metal-insulator (IMIMI) waveguide structure, and surface plasmon polariton (SPP) modes supported by a semi-infinite IMIMI waveguide were numerically solving an implicit dispersion relation. We found peaks in the amplification band in the optical transfer function were associated with IMIMI waveguide modes. After revealing this, the double-layer superlens imaging system was systematically designed by controlling SPP waveguide modes. We first analyzed two independent IMI with varying metal thicknesses from 5 nm to 100 nm, and their coupling effects when they got closer from 100 nm to 0 nm. Thin f-MIM, e.g., Al(5 nm)/SiO_2_(5 nm)/Al(5 nm), was suggested to enhance higher-spatial-frequency evanescent waves, with three amplification bands at $k_{x}/k_{0}$ = 1.7462, 6.0719, and 10.7102 associated with s-LRSPP, a-SRSPP, and s-SRSPP, respectively. The influence of the material index of the middle dielectric layer was then studied and found that the effective index $k_{x}/k_{0}$ of a-SRSPP and s-SRSPP increased to 7.7989 and 17.3991, respectively, when material (SiO_2_ with *n* = 1.55) was replaced by MgO with *n* = 2.02. Although higher-spatial-frequency evanescent waves were enhanced, the high peak of transfer function associated with the s-LRSPP at lower-spatial-frequency was detrimental to imaging. We discussed the completely s-LRSPP cutoff method and an approach to the s-LRSPP cutoff method. Assisted by approaching to the s-LRSPP mode cutoff method, the material index of object space was chosen as the commonly used LuAG with *n* = 2.14 to trim off the sharp amplification band, which created a flat and broad transfer function of the imaging system. The improved subwavelength imaging performance by double-layer metal superlens was validated by imaging an ideal thin object of two slits with 20 nm width distanced by a 20 nm spacer. The designed imaging system with double-layer superlens could further achieve a resolution limit of at least ~λ/16 for an isolated two-slit model under 193nm illumination. Our work provided sound theoretical analysis and a systematic design approach of double-layer metal superlens for near-field subwavelength imaging, such as fluorescent micro/nanoscopy or plasmonic nanolithography.

## Data Availability

Data underlying the results presented in this paper are not publicly available at this time but may be obtained from the authors upon reasonable request.
